# The Reaction Switching Produces A Greater Bias to Prepotent Response than Reaction Inhibition

**DOI:** 10.3390/brainsci10030188

**Published:** 2020-03-24

**Authors:** Kirill Fadeev, Tatyana Alikovskaia, Alexey Tumyalis, Alexey Smirnov, Kirill Golokhvast

**Affiliations:** 1 NTI Center for Neurotechnology and VR/AR Technologies, Far Eastern Federal University, Vladivostok 690922, Russia; fadeevk.fefu@gmail.com (K.F.); smirnov.aserg@dvfu.ru (A.S.); 2 Far Eastern Scientific Center of Russian Academy of Education, Far Eastern Federal University, Vladivostok 690922, Russia; alikovskaia.ta@dvfu.ru (T.A.); golokhvast.ks@dvfu.ru (K.G.); 3 Pacific Geographical Institute FEB RAS, Vladivostok 690014, Russia

**Keywords:** errors, reaction time, response inhibition, response switching

## Abstract

There is a discussion about common or various mechanisms of response inhibition and response switching. To understand these mechanisms, we used a modified Go/NoGo task with three stimulus categories. The subjects were instructed to press a button in response to frequent Go stimuli, press another button in response to rare Go stimuli and hold any motor response following the presentation of NoGo stimuli. The results showed a decrease in reaction time for frequent Go, following both categories of rare stimuli and the decrease was greater following rare Go. Also, the total number of errors did not differ between Go and NoGo, however, a greater bias of error rate towards frequent Go stimuli was found for rare Go compared to NoGo. Finally, positive correlations were found between the increase in reaction time for rare Go compared to frequent Go and the number of errors for both rare Go and rare NoGo. Together, these results indicate that both rare Go and NoGo stimuli required to inhibit the prepotent response, but rare Go in comparison to NoGo stimuli also evoked a conflict between prepotent and alternative responses, which is expressed in greater response bias toward frequent Go.

## 1. Introduction

Behavior flexibility includes both reaction inhibition and reaction switching, however, relationship between them is not clear. There are different opinions about its underlying mechanisms. Some authors [1,2,3] claim the existence of a common mechanism for inhibiting and switching reactions. Other researchers [4,5,6] suggest the existence of separated mechanisms. Also, inhibition is considered as a particular stage in response switching to cancel out an inappropriate response [7,8].

The Stop-Signal task (SST) and the Go/NoGo are the most used tasks to study response inhibition. In the SST, the presentation of a Go stimulus is followed after a short delay by a Stop stimulus, which signals that the execution of a reaction must be stopped. In the Go/NoGo task, a Go stimulus requires a subject to perform a motor response, and for a NoGo stimuli any reaction must be halted. Despite the existence of a high correlation within the number of errors between the SST and the Go/NoGo tasks [4], there are also differences in the specific mechanisms of the reaction’s implementation. In SST, the Stop stimulus is presented after the Go stimulus with variable delay, i.e. the inhibition of an already initiated reaction program occurs. In the Go/NoGo task, a separate NoGo stimulus (or Stop signal with zero delay), to which the inhibition is associated, initiates the inhibition of reaction [9].

To explain these reaction inhibition mechanisms Horse-Race Model and a Diffusion Decision Model were developed.

According to the Horse-Race Model (HRM) [10], the presentation of the stimulus starts two competing processes i.e. activation and inhibition. The process that first reaches the threshold of motor response wins and produces the effect of the presence or absence of a motor response.

The Diffusion Decision Model (DDM) was originally developed for two-choice decision tasks [11], however, it has been modified for Go/NoGo task [12,13]. According to this model, the threshold of motor response is achieved through the evidence accumulation from various sources (proprioception, interoception, and exteroception). Thus, the presence of the motor response is based on reaching evidence for its execution, and for NoGo stimuli there is the latent threshold for motor inhibition, which changes in a similar way to an actual threshold. 

Attention contributes greatly to the success of motor reaction inhibition. Since the No-Go stimuli are rare, they trigger an orienting response, which is the reaction on an unexpected stimulus in the form of inhibition of current activity, activation of the sensory system, and subsequent decision-making on the nature of the reaction [14]. Among other indicators, an orienting response is manifested as an increase in the motor reaction time [15]. Multiple studies have found that reaction stopping and orienting response share a common neuronal mechanism [1,3]. However, the reaction time increase could be due to various mechanisms. Particularly, the response delay also could be produced by the response conflict between the prepotent response to a frequent stimulus and the requirement to switch the response to a rare stimulus with a subsequent conflict resolution by removing an inappropriate reaction [16].

To take a closer look at the mechanisms of attention influence on motor reactions, in some studies researchers added the third stimulus category, rare Go, similar in frequency to a Stop stimulus, but requiring the response execution. If the frequent and rare Go stimuli require to press the same button [17,18,19,20], than the response accuracy for rare Go reaches almost 100% and the increase in motor reaction time (RT) is small, but significant. For example, in the study by Sharp et al. [20], rare Go trials with the same button press show about 40 ms increase relative to frequent Go trials and more than 97% success rate. In the study by Erica-Florence et al. [19] the increase was 20 ms for the inhibition task and 26 ms for the SST. In the Liebrand et al. [21] study researchers parcellated motor response delay to attention and motor switching components by presenting to the participants the rare Go stimuli with the same button press as frequent Go and the other rare Go stimuli with the alternative button press. The authors found that the non-switching response produced a small reaction delay, reflecting the attention mechanism, but the response switching produced additional delay, indicating the involvement of the motor regulation mechanism of an alternative response initiation. 

Verbruggen et al. [5] considered the question whether involved processes run in parallel or sequential using the task with the sequential performance of two responses. The results speak in favor of the sequential process, when activation of the first Go response should be inhibited and replaced by the activation of the second response. These results were confirmed in the studies [22,23] during the selective inhibition of motor response using myography and transcranial magnetic stimulation. Based on these data, the Activation Threshold Model (ATM) [24] was developed. Unlike previous models, ATM assumes that there is a change in the response excitation and inhibition thresholds in different phases of motor response. In the first phase, a generalized neuronal activation produces an undifferentiated motor response. In the next phase, a global inhibition of the motor reaction increases the reaction threshold. If response inhibition is insufficient then an error is made, and, finally, the differential activation produces the selective motor response.

The ratio of inhibition speed of the first response and the initiation of an alternative reaction is investigated with the Stop-Change Task, the extended version of SST [1,7,25,26,27]. This task includes three categories of stimuli - frequent Go, Stop signal and Change signal and requires the execution of the response, cancel the response and execution of the alternative response respectively. Similarly to the Stop-Signal RT (SSRT), the Change-Signal RT (CSRT) is calculated, based on the staircase tracking algorithm. According to the sequential assumption, at least, the response switching speed should not differ from the response inhibition speed. In an opposite situation, initiation of alternative response occurs before completing the first response inhibition. As a result, the research data are not consistent. High positive correlation between CSRT and SSRT was found [7], suggesting the existence of shared mechanism [1]. In some studies, CSRT was greater than SSRT [7,8]. Other authors found greater SSRT, than CSRT [26,28]. In both latter studies, the Change-response buttons were the same as Go-response buttons. Although, these data seem to speak against the assumption of serial processes in Change trials, however, it could be interpreted in this viewpoint. Particularly, according to ATM, initial Go-stimuli activates the undifferentiated response, which, perhaps, is not completely inhibited, and facilitates an alternative response, producing faster CSRT than SSRT. The opposite view is that the parallel activation of the motor programs for alternative responses could compete with each other and decrease the change speed.

Thus, task settings affect the response change speed and determine the association between stimulus and response. The results by Gordi et al. [25] shed light on this issue. They found that additional information about the response increases the response change speed in event-related design. However, in block design the response change speed were similar in trials with and without information about the response. Hence, task settings increase the speed or reduce information impact on the response change speed.

The reaction time to Go stimuli following Stop or Change stimuli is the additional parameter to evaluate the differences between the response inhibition and the response change. In the study by Verbruggen & Logan [29] participants performed the Stop-Change task. In Go1 trials participants responded by the right hand; in Change trials the Go2 stimuli were presented after the Go1 stimuli with some delay and subjects had to inhibit the response by the right hand and execute alternative response by the left hand; in Dual task trials they were instructed to respond to both Go1 and Go2 stimuli by the right and left hands respectively. The authors found that RT for Go2 stimuli were around 100 ms faster for Change trials than for Dual task trials. This indicates that in Change trials subject successfully inhibited the right-hand response, but in dual task the response programs for Go1 and Go2 trials competed with each other, producing longer RT. In the following Go1 trials subjects showed the opposite results. The Go1 after the Change trials produced greater reaction time increase, than the followed Dual task. The authors gave two explanations for this result. First, Go1 response was active in Dual task trials and could be easier to reactivate in the next trial, compared to Change trials, where Go1 response needed to be inhibited and was harder to activate in the next trial. Second, response selection was complicated with two goals (Go2 and Stop) in Change trials and was simpler in Dual task trials. Also, we assume, that the effect can be interpreted as a result response hand. In Change trials subjects inhibited the right-hand response, and in the next trial they had to return to the right hand responding. The Dual task trials required the responses by both hands, so in the next trial Go1 reactivation of the response were easier and RT delay were smaller.

The other results were found in the study by Krämer et al. [26]. They compared the reaction times for Go stimuli prior compared to follow Stop and Change trials and found that RT for Go stimuli was increased after correct Change trials and was decreased after the correct Stop trials. The different results in these studies could be due to the difference in paradigm. In the Verbruggen & Logan [29] there was no separated stop response condition, but only change trials, assuming that the stop process is a component of response switching. In Krämer et al. [26] study, participants performed the Eriksen flanker task with Go and Stop trials, complicating the interpretation of the results. Thus, the mechanisms, determining the differences between RTs for the Go stimuli followed by stop or switch stimuli are not clear.

Thus, up to date research results in the field of behavior mechanisms of response inhibition and switching are inconsistent and required further researches. In the present study, three types of stimuli, which differed in frequency and the requirement for a motor reaction, were presented to investigate the shared and different mechanisms between inhibition and switching of a motor response. A frequent Go stimulus required a subject to press the keyboard button, a rare Go stimulus was associated with pressing another keyboard button, and for NoGo stimuli subjects were instructed to refrain from making any motor response. If inhibition and switching of reactions have a common mechanism, then the success rates for the rare stimuli (rare Go and rare NoGo) could not be distinguished. If they are based on various mechanisms, then the reaction accuracy could vary depending on the difficulty of inhibiting or switching the reaction. Since two types of rare stimuli were used, we also had the opportunity to divide the errors into two categories–towards the frequent Go stimulus and towards another rare stimulus and assess the difference between rare Go and rare NoGo stimuli in these error subcategories.

The second goal of the study was to analyze the relationship between the increase in RT for the rare Go compared to the frequent Go, switching cost, and the number of errors for the rare stimuli. We analyzed the number of errors, rather than the number of correct avoidances, because the errors in the design of this experiment could be divided into subcategories. Since switching cost includes inhibition of an inappropriate reaction, we expected a negative correlation with the number of errors on NoGo stimuli. Alternatively, a greater increase in reaction time may be due to the conflict between a predisposed responding to a frequent Go and the requirement to change the reaction when a rare Go is presented, therefore the probability of errors would increase, i.e. the number of errors would have a positive correlation with the increase in reaction time for a rare Go stimuli.

## 2. Materials and Methods

### 2.1. Subjects

The subjects were 112 students and the staff from Far Eastern Federal University. Data for four subjects were removed from the analysis due to significant deviations in age. Three subjects had low NoGo success and very fast reaction times for the frequent Go, so we further removed their data from the analysis. The remaining 105 subjects (Mean ± Standard Deviation, age = 20.71 ± 2.31 years, body mass index = 21.96 ± 3.14, education = 14.01 ± 3.16 years, 52 males) refrained the use of energy drinks before the test, did not drink alcohol the day before the study and slept at least 6 hours a night before participating in the study. All subjects were volunteers, did not suffer from any mental or neurological disorders according to the self-report. All subjects gave their informed consent for inclusion before they participated in the study. The study was conducted in accordance with the Declaration of Helsinki, and the protocol was approved by the FEFU Ethics Committee.

### 2.2. Procedure

The subjects sat on a chair in front of a 24” monitor, connected to a PC with Intel Core i7-7700, 16 GB RAM, NVIDIA GeForce 1080. The distance between the subject and the screen was about 60 cm. Three types of stimuli were presented in the task – one frequent and two rare. For the frequent Go the subjects should press the left arrow button on the keyboard and for the rare Go the subjects should press the right arrow button. For the NoGo stimuli the subjects should refrain from pressing any button. A square was used as a frequent Go stimulus, a circle was used as a rare Go, a triangle was used as a rare NoGo. The figures were presented on a black background, were in gray and the size was 5x5 cm. A total of seven hundred stimuli were presented, 70% (490 stimuli) of which were frequent Go, 15% (105 stimuli) were rare Go, and 15% (105 stimuli) - NoGo. The task was divided into four blocks, having 175 stimuli per block, with a 1–2 min rest interval between blocks. The total duration of the experiment was about 45 min, including questionnaire, informed consent, instructions, training, pauses between the trials and the task, which was about 21 min in duration. The sequence of stimuli presentation was made according to the rules:

1. Before and after each rare stimulus, there should be frequent stimuli;
2. Rare stimuli from the same category (rare Go or NoGo) were presented no more than two times in a row, separated by frequent stimuli;
3. To reduce the subjects’ expectations (because after a rare stimulus there is always a frequent Go), two mini-sequences, consisting of two rare stimuli in a row were inserted into each block. The subjects’ reactions to stimuli in these mini-sequences, as well as reactions to frequent Go stimuli before and after each mini-sequence were excluded from the analysis;
4. Each block began with a frequent Go stimulus, reactions to which were excluded from the analysis.

During a trial, a gray cross appeared in the center of the screen with randomly varying duration from 500 to 700 ms with 50 ms step, followed by a stimulus for 200 ms, and then the subject had one-second window to respond by pressing the corresponding button. The interval between trials was randomly set from 1 to 1.5 sec with 100 ms step. The task instructions asked the subjects to focus on the speed and ignore the reactions to errors made.

Further, all stimuli were divided into the following categories:

1. Rare Go stimuli (rGo);
2. Rare NoGo stimuli (NoGo);
3. Frequent Go stimuli before rare Go stimuli (rGo-1);
4. Frequent Go stimuli after rare Go stimuli (rGo+1);
5. Frequent Go stimuli before rare NoGo stimuli (NoGo-1);
6. Frequent Go stimuli after rare NoGo stimuli (NoGo+1);
7. Frequent Go stimuli between other Go stimuli (fGo).

Each category of rare stimuli was divided into three groups—correct answers and two types of errors. For a NoGo stimulus there were two possible errors towards a frequent Go or rare Go stimulus. For a rare Go stimulus there were errors towards frequent Go or signal omission.

The number of errors was calculated for the following categories:

1. Errors towards frequent Go for rare Go stimuli (rGo_to_fGo);
2. Missing the reaction for rare Go stimuli (rGo_to_NoGo);
3. Errors towards frequent Go for rare NoGo stimuli (NoGo_to_fGo);
4. Errors towards a rare Go for rare NoGo stimuli (NoGo_to_rGo);
5. The total number of errors for rare Go stimuli (rGo, total);
6. The total number of errors for NoGo stimuli (NoGo, total).

Finally, the reaction time (RT) for the correct responses to stimuli was analyzed. There was not enough data to analyze the RT for errors, so, reaction times were calculated only for correct responses. Reactions less than 150 ms were deleted as premature. Further, the standard deviation of RT was calculated for each category of stimuli, and reactions exceeding three standard deviations were removed. The remaining reactions were averaged separately by categories.

The distribution of mean RT and the number of errors among the subjects was asymmetrical. To perform parametric tests, the RT of each subject was logarithm transformed with a natural base, and the square root of the percent number of errors for each subject for each category of errors is calculated. The resultant transformation of the RT data and the percent number of errors did not differ from the normal distribution (according to the Kolmogorov-Smirnov test).

### 2.3. Statistical Analysis 

To analyze the RT for frequent Go stimuli subcategories, we used ANOVA with five levels (fGo, rGo-1, rGo+1, NoGo-1 and NoGo+1). We were interested to know whether there is a general effect of the difference in RT depending on the position of the frequent Go. Next, we compared the RT for fGo with the RT to other subcategories of frequent Go. For this, we used post-hoc comparisons of mean values using the Bonferroni correction.

Further, RT for subcategories of frequent Go before and after rare stimuli were subjected to ANOVA with factors of rare stimulus Category (rare Go, NoGo) and Time (Before and After). Post-hoc comparison of mean values was performed using the Bonferroni correction.

The total number of errors and the number of errors towards frequent Go for rare Go and NoGo were compared using the Student *t*-test. The number of errors towards rare Go for NoGo and the number of reaction omissions for rare Go had a non-normal distribution and were compared using the nonparametric Wilcoxon matched pairs test method.

To analyze the magnitude of the effect, a partial η squared was used. When performing ANOVA, the Greenhouse–Geisser correction was used to control sphericity, where necessary.

The Spearman correlation coefficient was used to analyze the relationship of variables.

The significance threshold value was set at p < 0.05. 

## 3. Results

### 3.1. Reaction Time

**Figure 1:**
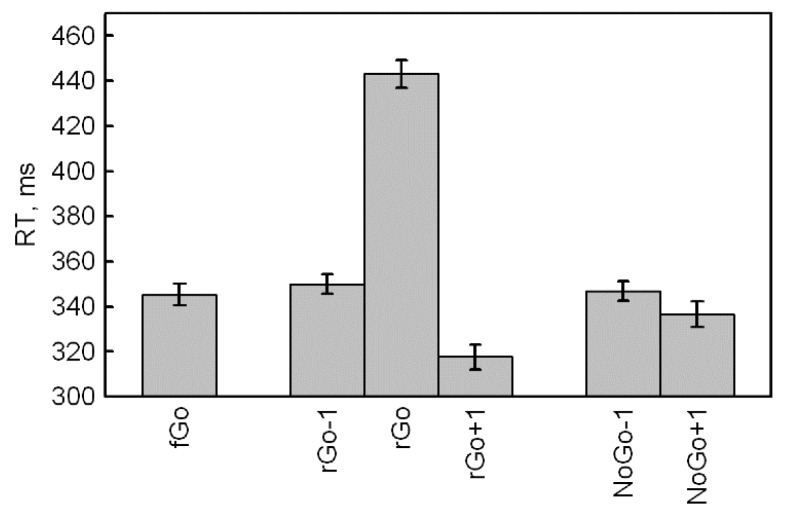
Reaction times for rare Go and subcategories of frequent Go stimuli.

First, we were interested in RT differences for frequent Go stimuli, depending on its position relative to rare stimuli. An ANOVA included five Categories of frequent Go (fGo, rGo-1, rGo+1, NoGo-1 and NoGo+1) and a significant effect of Category (F(4,416) = 80.0, *p* < 0.001, $\eta_{p}^{2}$ = 0.435, ε = 0.563) was found. Our results demonstrate that RT for fGo does not differ from rGo-1 (*p* = 0.294, Bonferroni correction) and NoGo-1 (*p* = 1.000, Bonferroni correction). We found that RT for fGo also was longer as compared to rGo+1 (*p* < 0.001, Bonferroni correction) and NoGo+1 (*p* < 0.001, Bonferroni correction) (Table 1 and Figure 1). 

Second, an additional ANOVA was performed for four subcategories of frequent Go before and after rare Go and NoGo and included the factors Category (rGo, NoGo) and Time (Before, After). The results showed significant main effects of the stimulus Category (F(1,104) = 43.9, *p* < 0.001, $\eta_{p}^{2}$ = 0.297, ε = 1.000), Time (F(1,104) = 98.9, *p* < 0.001, $\eta_{p}^{2}$ = 0.487, ε = 1.000) and the interaction Category × Time (F(1,104) = 64.9, *p* < 0.001, $\eta_{p}^{2}$ = 0.384, ε = 1.000). The effect of the stimulus category indicated that the response time for frequent Go stimuli before and after rare Go was faster compared to RT for frequent Go stimuli before and after NoGo. The time effect indicated that the frequent Go stimuli before both categories of rare stimuli (rGo and NoGo) have a longer RT as compared to RT to frequent stimuli after both categories of rare stimuli. Finally, the interaction effect revealed that the RT for frequent Go stimuli before rare ones (rGo-1 and NoGo-1) did not differ (*p* = 0.885), but were significantly faster after both categories of rare stimuli (all *p* < 0.001). Also, the RT for rGo+1 was faster than the reaction time for NoGo+1 (*p* < 0.001, Bonferroni correction).

To analyze the RT increase for rGo, we used the RT for frequent Go before and after rare Go and the reaction time for rare Go stimuli (rGo-1, rGo, rGo+1). A significant stimulus effect was found (F(2,208) = 810.1, *p* < 0.001, $\eta_{p}^{2}$ = 0.886, ε = 0.895), indicating a longer RT for rGo compared to rGo-1 and rGo+1 (all *p* < 0.001, Bonferroni correction).

Thus, according to the RT data, frequent Go stimuli among other frequent Go did not differ from frequent Go before rare stimuli. However, frequent Go stimuli after rare ones had shorter reaction time and a greater decrease was found after rGo compared to NoGo. Also, rare Go stimuli showed longer RT compared to frequent Go stimuli before and after rare Go.

### 3.2. Reaction Accuracy

**Figure 2:**
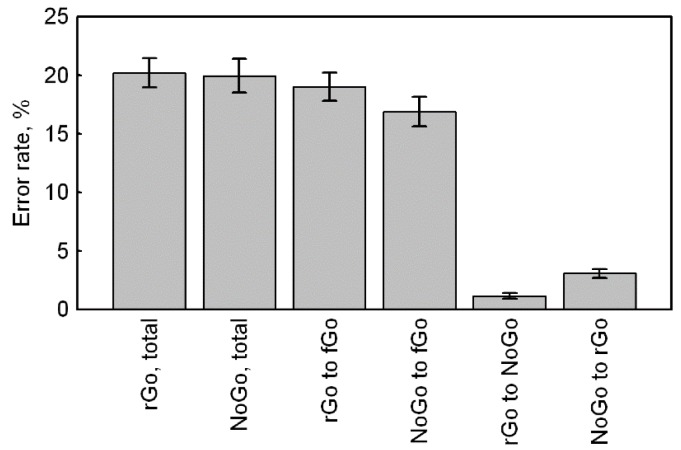
The error rates for total number and subcategories of errors for rare Go and NoGo stimuli.

To analyze the response accuracy, we used the data of the total number of errors for rGo and NoGo stimuli, the number of errors towards frequent Go for rGo and NoGo stimuli, the number of errors towards rare Go for NoGo stimuli, and the signal omissions for rare Go stimuli (Figure 2). The square root of error rates for all variables were calculated in order to achieve normal distribution (Table 2). Since the number of omissions for rGo included a lot of zero values, all comparisons involving this variable were performed using non-parametric criteria. The remaining comparisons were performed using the Student’s t-test.

The total number of errors for rare Go and NoGo did not differ from each other (t (104) = 1.09, *p* = 0.278, CI = −0.09–0.30).

The number of errors for rare Go toward frequent Go were greater than the number of errors for NoGo toward frequent Go (rGo_to_fGo versus NoGo_to_fGo: t (104) = 3.45, *p* = 0.001, CI = 0.14–0.51).

The number of omissions for rare Go were less in comparison with the number of errors for NoGo toward rare Go (rGo_to_NoGo versus NoGo_to_rGo: Z (83) = 4.95, *p* < 0.001).

The number of errors for NoGo toward frequent Go was greater compared to the number of errors for NoGo toward rare Go (NoGo_to_fGo versus NoGo_to_rGo: t (104) = 16.15, *p* < 0.001, CI = 2.08–2.66).

Thus, a total number of errors was equal, but the rare Go stimuli evoked more errors towards the frequent stimuli, as compared to NoGo stimuli, and fewer omissions (errors towards NoGo), compared with errors towards a rare Go stimuli for NoGo.

### 3.3. Correlations

**Figure 3:**
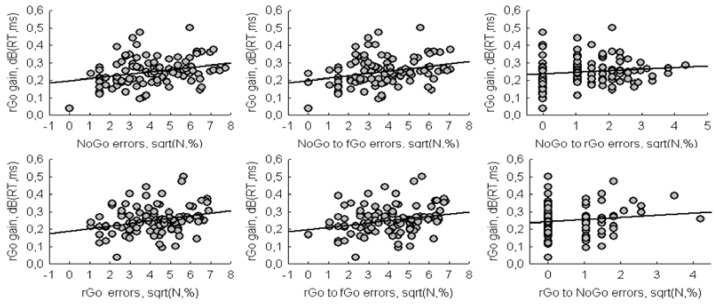
Scatterplots of correlations between the number of errors for rare Go and NoGo stimuli and RT gain for rare Go stimuli.

The second objective of the study was to analyze the relationship between switching response and response inhibition. We used the number of errors for NoGo as an indicator of the failure of motor inhibition, and the increase in RT for rGo as an indicator of switching cost. The increase in RT for rGo was calculated as the difference between the logarithmic values of RT for rGo and fGo. The difference in the logarithms of the two variables is known as Decibels. Since inhibition is involved in both processes, based on the assumption of Boecker et al. [1] and Wessel and Aron [3], it was expected to find a significant negative correlation between these two variables. However, the significant positive correlation (r = 0.27, *p* = 0.005) was found, i.e., an increase in RT gain for rare Go was accompanied by an increase total errors for NoGo (Figure 3). The correlation between the increase in RT for rGo and the total number of errors for rGo was also significant (r = 0.29, *p* = 0.003).

In addition, there were significant correlations between rGo gain and the number of errors for rare stimuli toward frequent Go (NoGo_to_ fGo: r = 0.28, p = 0.004; rGo_to_ fGo: r = 0.25, *p* = 0.009). The correlations between rGo gain and the number of errors for NoGo toward rGo (Pearson r = 0.12, *p* = 0.214) as well as between rGo gain and the number of errors for rGo toward NoGo (Spearman r = 0.11, *p* = 0.268) were non-significant.

## 4. Discussion

In the present study, we investigated whether behavior inhibition and switching have common or different mechanisms. For this purpose, we used modified Go/NoGo task with frequent Go, rare Go and rare NoGo stimuli. It was found, that the RT for frequent Go stimuli after both categories of rare stimuli was shorter than the RT for frequent Go stimuli before rare stimuli, and a greater reaction speed was found after rare Go compared to NoGo. It was also found that although the total number of errors for rare Go and NoGo did not differ, rare Go showed the greater error bias towards a frequent Go stimulus than NoGo. Positive correlations were found between the RT gain for rare Go and the total number of errors and errors rate toward frequent Go for both rare Go and NoGo stimuli.

In the literature, both general [1,7] and different [4,5] mechanisms for response inhibition and response switching have been reported. Neurophysiological data also are not consistent. Sebastian et al. [30] has shown, that various regions of the frontal cortex are involved in the regulation of motor reactions for rare Go and NoGo stimuli. On the contrary, Wessel and Aron [3] suggested the presence of only one mechanism for both the orienting reaction to rare stimuli and response inhibition. The current study is focused on behavioral effects of response inhibition or switching for the rare stimuli.

The effect of the position of a frequent Go stimulus on RT was previously discovered in a study by Cheyne et al. [31]. They have found the increase in the RT before and the decrease after NoGo. According to these findings, we also separated all frequent Go stimuli into subcategories: frequent Go before and after rare stimuli and frequent Go between other frequent Go. RT data showed that frequent Go between other frequent Go and before rare Go and NoGo did not differ. These results do not correspond to the data from Cheyne et al. [31]. The difference could be due the fact that their study had a much larger sample size (504 subjects), which lowered the threshold for statistical significance of differences in mean values. Also, in the Cheyne et al. [31] study design the NoGo stimuli were less frequent, so the subjects showed the increased RT for frequent Go before rare stimuli, reflecting their caution in the expectation of a NoGo.

There was a decrease in response time for frequent Go after rare stimuli (Table 1 and Figure 1). We suppose that this is an effect of anticipation, as fGo always followed rare stimuli [32]. However, frequent Go following rare Go showed faster RT compared to RT for frequent Go after NoGo.

The response preparation [16] and response execution [32] affected the response to the following stimuli, prolonging the RT. It seems conflicting with the current results, but the differences in methods should be mentioned. In their research subjects performed the response set switching task, but in the current experiment response set was constant. The greater response bias toward the frequent Go for the rare Go in the current experiment should have evoked greater interference in the case of response set switching.

Based on these observations, the following three hypotheses can be put forward.

The first hypothesis is that following the NoGo stimulus, response inhibition extends to the next Go. Therefore, anticipation for response execution is superimposed on inhibition and, as a result, there is a slower RT following NoGo. For the frequent Go following rare Go inhibition is lower, since a motor response in the previous trial was required. However, Philipp et al. [32] did not find the significant effect of interstimulus interval duration on the RT deceleration for the next stimulus, which may be an argument against this hypothesis.

According to the second hypothesis, the reaction threshold for rare Go is closer to the reaction threshold for frequent Go than the implicit reaction threshold for NoGo. So, presentation of rare Go evokes a stronger expectation towards frequent Go.

The third hypothesis states that rare Go and NoGo were evoking varying degrees of conflict. For rare Go stimulus, the conflict exists between the response tendency toward frequent Go and the requirement to perform the alternative response, thus response inhibition tendency for NoGo is ignored. This assumption is supported by data from Hong et al. [33], which indicates that the ignored NoGo stimulus did not evoke the P300 event-related potential component as compared to attended NoGo. In turn, presentation of NoGo stimuli conflicts with two response tendencies - toward frequent Go and rare Go. So, the frequent Go following NoGo is presented in a context of a conflict with two response trends, but the frequent Go following rare Go is presented in a context of a conflict with only one response tendency. The situation was more certain for rare Go than for the NoGo and, therefore, the reaction time was faster.

The third hypothesis can be reduced to second hypothesis in the situation with a very high motor threshold for NoGo compared to both rare Go and frequent Go, minimizing the NoGo threshold influence [29].

Since the use of a modified Go/NoGo task allowed us to separate the errors into subcategories, we did not analyze the number of correct answers, but the number of errors instead. In this study, NoGo errors were divided into errors towards frequent Go and errors towards rare Go. Rare Go errors were divided into errors toward frequent Go and signal omissions, i.e., towards NoGo. Also, the total number of errors for rare Go and NoGo were analyzed.

We found that the total number of errors did not differ between rare Go and NoGo, despite the difference in the requirements to produce a motor reaction. First, the data may indicate the effect of attention on inhibition. This correlates with a wide field of data on the relationship between inhibition and attention [18,19,20,30,33,34,35]. Inhibition, as suggested by Schuch and Koch [16], acts as an element of conflict resolution, reducing the number of degrees of freedom in favor of the realization of an appropriate reaction. The data also correspond to the DDM on the existence of an implicit response threshold for NoGo stimuli. Second, the response switching is as effective as the response inhibition. Switching reaction required inhibition of the prepotent response followed the activated of a new reaction [1,8,24,29]. When a rare stimulus is presented and reaction should not be changed [17,18,19,20], the accuracy of execution is close to 100%. In the present study, the reaction for the rare Go changed compared to frequent Go, and the total number of errors was similar to the inhibited reaction. Therefore, the main factor that increases the number of errors is the requirement to inhibit a prepotent response and then to change a response. Ineffective inhibition of the conflicting responses causes the error, and whether the rare reactions is explicit or implicit does not affect the total number of errors. This data correspond to the ATM, since the total number of errors on rare stimuli was similar and, therefore, the general mechanism of motor response inhibition is involved, followed by the initiation of a new response with a longer RT to the rare Go. Thus, we assume that the increase in response time for rare Go in the present study has the common cognitive component of the orienting reaction for both the rare Go and NoGo, and an additional switching cost exists for rare Go.

The differences between two rare stimuli did not relate to the total number of errors, but to their distribution between the subcategories. The NoGo errors towards frequent Go stimuli 5.58 times exceeded the number of NoGo errors towards rare Go stimuli. The ratio between presentations of frequent Go and rare Go stimuli was 4.67. Therefore, response bias toward frequent Go was stronger than the objective ratio of stimuli, i.e., subjects exaggerated the frequency of frequent Go stimuli and/or downplayed the frequency of rare Go. Thus, the design of the present study produces a strong response bias toward frequent stimuli.

The number of errors towards frequent Go was greater for rare Go compared to NoGo. On the other hand, the number of errors towards rare Go for NoGo was greater than the number of signal omissions for rare Go. That is, rare Go errors were more biased towards frequent Go, and some NoGo errors were redistributed towards responses to rare Go. Thus, the presence of explicit motor reaction to a rare stimulus affected the shift of the reaction threshold towards a prepotent response. These results correspond to RT data for frequent Go following by rare Go and NoGo, and indicates the greater bias for rare Go, compared to NoGo, towards frequent Go. So, requirement to execute the motor response for the rare Go additionally primes the response tendency toward the frequent Go and, thus, the greater effort is needed to overcome the prepotent response.

The increase in RT for rare Go relative to frequent Go was obtained in the current study. This RT gain could be the result of an orienting response, which inhibits the current motor activity in favor of more rigorous sensory analysis of an unexpected stimulus [3]. It could be also the result of a motor conflict between the prepotent response, which should be inhibited, and the realization of the required reaction. Both variants involve the inhibition of the current reaction tendency, but the latter differs from the former in the presence of motor interference, resulting in increasing the error rate. Some authors [1,2,3] suggested the presence of a common mechanism for response switching and response inhibition. It could be concluded from the assumption that a greater increase in a motor RT for rare Go stimulus, relative to the RT to a frequent Go stimulus, produces a negative correlation with the number of errors. In other words, a greater inhibition leads to a greater increase in RT for rare Go and at the same time to fewer errors for NoGo stimuli. However, in the present study, positive correlations were found between the motor reaction time gain for a rare Go stimulus and the total number of errors as well as the number of errors towards frequent Go for both rare Go and NoGo stimuli. Correlations between RT gain for rare Go and number of errors toward other category of rare stimuli were non-significant. These results indicate that, response switching and response inhibition share some mechanisms, and RT gain for rare Go reflects the mechanism of overcoming the prepotent response, and support rather motor conflict assumption, than solely motor inhibition. So, a greater RT gain for rare Go occurs due to the competition of tendencies between performing prepotent responses toward frequent Go and requirement to switching response for rare Go, and a greater conflict lead to a greater likelihood of errors toward frequent Go.

If only a single response button for both the frequent Go and rare Go stimuli is used [17,18,19,20], then the small RT gain is found, which does not produce the response conflict and reflect the cognitive conflict between the expected and presented stimuli (orienting response) expressed in motor inhibition. Wessel and Aron [15] in their studies proceeded from the assumption that the RT gain is an indicator of orienting response (inhibition of current motor activity in favor to more precise sensory analysis of unexpected stimuli), but in their experiments, they actually used the response switching, including the response conflict between current motor activity and alternative response execution. In the current experiment, response switching results in the RT gain for the rare Go and associated with the motor conflict between the prepotent and alternative responses. The absence of the motor reaction for NoGo is associated with the redistribution of attention towards rare Go, and this decreases its association with the prepotent response to frequent Go and helps to switch the response set. This assumption is confirmed with the studies of Schuch and Koch [16] and Philipp et al. [32], which provide the data that the switch in the response set does not increase of RT if a NoGo stimulus was presented in the previous trial. The presentation of a Go stimulus in the previous trial induced an interference with the response set switching and prolonged the RT. The authors suggest that in the latter case the effect results from the conflict between response sets, but our findings suggest that this conflict could be due to the greater response bias for the rare Go towards frequent Go.

Thus, in the present study, we found weak, but significant correlations between the RT gain for rare Go and error rate toward frequent Go, indicating, that response switching and response inhibition share some common mechanism. Based on negative signs of these correlations, we proposed that inhibition appears as a part of a conflict resolution between prepotent response and alternative response execution. Also, we found the difference between rare Go and NoGo in RT for the following Go stimuli as well as the difference between rare Go and NoGo in error rates toward frequent Go, suggesting the existence of varying mechanisms. Particularly, if an alternative response is required, it produces the greater prepotent response bias toward frequent Go, expressed in a faster RT for frequent Go following rare Go and a greater error bias toward frequent Go. In opposite, if no response is required, the total error rate is similar, but some errors are redistributed toward rare Go. This indicates that, besides common features between response switching and response inhibition, still there is a difference, which should be taken into account in future researches.

The present research has limitations. First, the experimental design with two responses was used, but in real life conditions usually there are more choices. Thus, a greater response bias for the rare Go, which was found in the present study, could be associated with the poor choice of alternatives. Second, we found an asymmetry in error rates for rare Go and NoGo. For the rare Go, there was one alternative in the form of a reaction to frequent Go, for the NoGo there were two reaction alternatives – frequent Go or rare Go stimuli. Thus, rare stimuli were not in the same condition, which affected the RT and the number of errors. A more balanced design of the experiment could include NoGo stimuli of a greater significance, so that the signal omissions for the rare Go stimulus would have a greater importance for the subject. Other designs could include rare Go with an alternative reaction. Third, there were not enough errors in the study to analyze the error reaction times, which could provide the additional information about differences between response switching and response inhibition. Fourth, a major limitation of our study concerns the correlative nature of results without a computational modeling, which can predict the test data for both behavior inhibition and switching. However, we hope, that our results will contribute to understanding the relationship between response inhibition and switching.

## 5. Conclusion

In this study, a modified version of Go/NoGo task with frequent and rare Go, as well as rare NoGo stimuli was used to investigate the common and different mechanisms of response switching and response inhibition. The decrease in RT for frequent Go following the presentation of both categories of rare stimuli was found, but the faster RT was found for frequent Go following rare Go, comparing with frequent Go following NoGo. The error rate did not differ between Go and NoGo, however, a greater bias of errors towards frequent Go stimuli was found for rare Go, compared to NoGo. Finally, positive correlations were found between the increase in RT for rare Go compared to frequent Go and the number of errors for rare stimuli. Together, these results indicate that rare stimuli produce the conflict between the prepotent response to a frequent stimulus and the alternative response. This conflict is greater for rare Go compared with NoGo, because response execution produces a greater conflict with prepotent response.

## Figures and Tables

**Table 1:** Reaction times for frequent and rare Go stimuli.

	RT			ln(RT)		
	M	SD	Skewness	M	SD	Skewness
fGo	345	49	0.93	5.84	0.13	0.64
rGo-1	350	45	1.08	5.85	0.12	0.77
rGo	443	62	0.90	6.08	0.13	0.52
rGo+1	318	56	0.85	5.75	0.17	0.41
NoGo-1	347	45	1.13	5.84	0.12	0.82
NoGo+1	337	58	0.77	5.80	0.17	0.29

**Table 2:** The error rates for rare Go and NoGo stimuli.

	N			sqrt (N, %)	
	M (SD)	M (SD), C %	Skewness, %	M (SD)	Skewness
rGo, total	18.15 (11.49)	20.20 (12.75)	0.45	4.23 (1.52)	−0.17
NoGo, total	17.96 (13.37)	19.97 (14.84)	0.76	4.12 (1.73)	0.07
rGo to fGo	17.11 (11.21)	19.05 (12.44)	0.46	4.08 (1.56)	−0.24
NoGo to fGo	15.20 (11.74)	16.89 (13.04)	0.86	3.76 (1.67)	0.01
rGo to NoGo	1.04 (2.18)	1.15 (2.42)	4.41	0.64 (0.86)	1.42
NoGo to rGo	2.76 (3.20)	3.09 (3.58)	1.80	1.38 (1.09)	0.27
